# Curcumin and Quercetin-Loaded Lipid Nanocarriers: Development of Omega-3 Mucoadhesive Nanoemulsions for Intranasal Administration

**DOI:** 10.3390/nano12071073

**Published:** 2022-03-25

**Authors:** Gustavo Richter Vaz, Mariana Corrêa Falkembach Carrasco, Matheus Monteiro Batista, Paula Alice Bezerra Barros, Meliza da Conceição Oliveira, Ana Luiza Muccillo-Baisch, Virginia Campello Yurgel, Francesca Buttini, Félix Alexandre Antunes Soares, Larissa Marafiga Cordeiro, Flavia Fachel, Helder Ferreira Teixeira, Juliana Bidone, Patrícia Diaz de Oliveira, Fabio Sonvico, Cristiana Lima Dora

**Affiliations:** 1 Graduate Program in Health Sciences, Federal University of Rio Grande, Rio Grande 96203-900, Brazil; richtervaz@gmail.com (G.R.V.); mari_falkembach@hotmail.com (M.C.F.C.); mbmatheus54@gmail.com (M.M.B.); alicebarros.pb@gmail.com (P.A.B.B.); melizacoliveira@hotmail.com (M.d.C.O.); anabaisch@gmail.com (A.L.M.-B.); virginia.yurgel@gmail.com (V.C.Y.); 2 Food and Drug Department, University of Parma, 43124 Parma, Italy; francesca.buttini@unipr.it (F.B.); fabio.sonvico@unipr.it (F.S.); 3 Graduate Program in Biological Sciences, Federal University of Santa Maria, Santa Maria 97105-900, Brazil; felix@ufsm.br (F.A.A.S.); larissa.marafiga@hotmail.com (L.M.C.); 4 Graduate Program in Pharmaceutical Sciences, Federal University of Rio Grande do Sul, Porto Alegre 90610-000, Brazil; flavia_fachel@hotmail.com (F.F.); helder.teixeira@ufrgs.br (H.F.T.); 5 Center of Chemical, Pharmaceutical and Food Sciences, Federal University of Pelotas, Pelotas 96010-610, Brazil; julianabidone@gmail.com; 6 Department of Biotechnology, Federal University of Pelotas, Pelotas 96010-610, Brazil; bilicadiaz@yahoo.com.br

**Keywords:** nanoemulsions, curcumin, quercetin, intranasal administration, toxicity

## Abstract

Curcumin (CUR) and quercetin (QU) are potential compounds for treatment of brain diseases such as neurodegenerative diseases (ND) because of their anti-inflammatory and antioxidant properties. However, low water solubility and poor bioavailability hinder their clinical use. In this context, nanotechnology arises as a strategy to overcome biopharmaceutical issues. In this work, we develop, characterize, compare, and optimize three different omega-3 (ω-3) fatty acids nanoemulsions (NEs) loaded with CUR and QU (negative, cationic, gelling) prepared by two different methods for administration by intranasal route (IN). The results showed that formulations prepared with the two proposed methods exhibited good stability and were able to incorporate a similar amount of CUR and QU. On the other side, differences in size, zeta potential, in vitro release kinetics, and permeation/retention test were observed. Considering the two preparation methods tested, high-pressure homogenization (HPH) shows advantages, and the CQ NE- obtained demonstrated potential for sustained release. Toxicity studies demonstrated that the formulations were not toxic for *Caenorhabditis elegans*. The developed ω-3 fatty acid NEs have shown a range of interesting properties for the treatment of brain diseases, since they have the potential to increase the nose-to-brain permeation of CUR and QU, enabling enhanced treatments efficiency.

## 1. Introduction

Polyphenols are substances that present antioxidant properties as a consequence of their free radical scavenging action and metal chelating ability, preventing the enzymatic production of reactive oxygen species catalyzed by metals and thus displaying neuroprotective effects [1]. These compounds, in particular flavonoids, when administered in animal models, are capable of enhancing synaptic plasticity [2] and are able to reduce the accumulation of neuropathological proteins [3]. Therefore, two natural compounds such as curcumin (CUR) and quercetin (QU) have been proposed as potentially therapeutic compounds for the treatment of brain disorders such as neurodegenerative diseases (ND) [4,5].

CUR is a polyphenol that has shown several potential therapeutic properties in numerous studies. Among the properties investigated, antitumoral [6], anti-inflammatory [7], and antioxidant activities [8] emerged as particularly relevant. However, despite the positive results of many studies in vitro and in some animal models, the clinical potential of CUR is very limited, as this compound shows poor oral bioavailability due to its low aqueous solubility, rapid intestinal and hepatic metabolism, and fast systemic elimination [9]. Similarly to CUR, QU has anti-inflammatory, antioxidant, and anti-carcinogenic actions [10]. QU also has neuroprotective activity and reduces the inflammation induced by cholesterol oxidation products that have been demonstrated to be a risk factor for neurodegenerative diseases [11,12]. However, these properties are limited by poor oral bioavailability and scarce distribution in the organism as a whole and to the brain [13]. 

The high lipophilicity of these compounds makes their therapeutic use a biopharmaceutical challenge [14,15]. In order to harness the clinical potential of these compounds, a suitable pharmaceutical form appears necessary to preserve chemical stability and increase bioavailability. Several formulation strategies have been described in the literature to enable the therapeutic use of compounds with such characteristics. Modifications of the vehicle pH, use of cosolvents, and the formation of cyclodextrins [16] are just a few examples to solubilize water-insoluble drugs for oral and injection administration. These approaches, however, show as main limitations: pain reports upon injection, possible precipitation of the drugs during the administration, and problems of biocompatibility among the excipients and/or with the active compound in the formulation. 

On the other hand, some studies have proven that CUR and QU, when nanoencapsulated, have some significant benefits if compared to their free form [17,18]. These benefits occurred mainly due to increased bioavailability and consequently higher therapeutic potential, either as immunomodulatory, neuroprotective, anti-inflammatory, or antioxidant treatments [17,19,20,21,22]. In this context, nanostructured carrier systems have been proved to be a promising formulation strategy. Among nanocarriers, lipid nanoemulsions (NEs) have been widely employed for drug delivery systems [18,23]. 

NEs are heterogeneous systems in which one fluid is dispersed in another non-miscible liquid in the presence of one or more emulsifying agents. Several studies have described as advantages of pharmaceutical NEs: reduction of toxicity, increase of therapeutic efficacy, enhancement of bioavailability, and, in some cases, control of the release of the compounds incorporated in these delivery systems [24,25,26]. The biocompatibility of the excipients used in the production of these nanocarriers makes them promising systems for administration via several routes [24]. Among the biocompatible lipids that can be used for NE production, docosahexaenoic acid (DHA) is particularly interesting for formulations with brain delivery goals. DHA is present in the neural membranes of the cerebral cortex and retina [27]. Delivering DHA to the central nervous system becomes interesting, because it is directly related to the membrane excitation [28], memory operation [29], neural signalization [30], function of photoreceptor cells [31], and in the neuroprotection [32,33]. In several NDs, a depletion of the ω-3 polyunsaturated fatty acids (PUFAs), directly related to the neuroinflammation, is often evidenced [34,35].

The administration of nanocarriers through alternative routes appears to be an interesting complement to nanotechnology in improving effectiveness and bioavailability of the compounds at specific sites of the organism. The intranasal (IN) route provides direct and non-invasive access to the brain, avoiding the blood–brain barier (BBB), increasing the amount of the compound reaching the central nervous system, and decreasing the side effects. Moreover, the IN route is painless and patient-friendly and improves the drug performance [36]. To use the IN route as an alternative access route to the central nervous system, the incorporation into the formulation of gelling agents appears to be an interesting approach to prolong residence time at the absorption site. In situ gelifiers, such as gellan gum (Gelzan™ CM), allow the nanocarrier formulation to change its viscosity only when in contact with the ions present in the physiological fluids, a phenomenon known as in situ gelation, which increases the viscosity of the formulation at the site of deposition, aiming to promote a more effective absorption of the active compounds [37,38]. 

Thus, the access to the central nervous system through the IN route using NEs containing in situ gelling agent appears to be an interesting possibility despite limitations such as the reduced volume of administration of the formulation and the characteristic mucociliary clearance of the nasal mucosal secretions. In this context, the present work aims to develop, characterize, compare, and optimize omega-3 (ω-3) fatty acid mucoadhesive NEs loaded with CUR and QU for administration by IN route.

## 2. Materials and Methods

### 2.1. Materials

Curcumin (CUR), quercetin (QU), cetalkonium chloride, deacetylated gellan gum (Gelzan™ CM), polyethylene glycol stearate 660 (Solutol HS15^®^), castor oil, and porcine stomach mucin (type II) were bought from Sigma–Aldrich (St. Louis, MO, USA). Egg phospholipids with 80% phosphatidylcholine (Phospholipon E80^®^) and purified fish oil (DHA/EPA) were obtained from Lipoid (Ludwigshafen, Germany). Acetonitrile of high-performance liquid chromatography (HPLC) grade was purchased from Panreac (Barcelona, Spain), and the pure water was achieved with a Milli-Q^®^ water system (Millipore, Burlington, MA, USA). Polyethylene glycol 400 (PEG400), potassium chloride, and sodium chloride were obtained from Synth (São Paulo, SP, Brazil). Monobasic sodium phosphate and dibasic sodium phosphate were purchased from Alphatec (Porto Alegre, RS, Brazil). The calcium chloride dihydrate was purchased from Vetec (Rio de Janeiro, RJ, Brazil). Ethanol, acetone, phosphoric acid, and other reagents were of analytical grade.

### 2.2. Lipid Nanocarriers Preparation

The hot solvent diffusion (HSD) method [39] and the high-pressure homogenization (HPH) technique [40] were combined with the phase inversion temperature technique to produce the nanocarriers as described in the following sections. The phase inversion temperature technique consists in a characteristic of PEG 660-stearate that becomes gradually lipophilic and migrates within the oily phase with the increase in temperature (~80 °C), reducing the size of the nanocarriers.

### 2.3. HSD Method Combined with the Phase Inversion Temperature Technique

In brief, a solution containing castor oil, Lipoid^®^ purified fish oil (DHA/EPA), and egg lecithin (Lipoid E80^®^) in acetone:ethanol (60:40, *v/v*; 2 mL) and 3 mL of acetone:ethanol (60:40, *v/v*), maintained at 68 °C, were added to an aqueous phase (50 mL) containing PEG 660-stearate surfactant (1.5% *w/v*) previously heated at 80 °C, under magnetic stirring, at 700 rpm. The colloidal suspension was then cooled to room temperature (±25 °C). The organic solvent was evaporated under reduced pressure in a rota-evaporator (Fisatom 803, São Paulo, SP, Brazil) at 23 mbar (~15 min), and the final volume was adjusted with Milli-Q^®^ water to 20 mL to obtain the blank NE (NEdif-). Lastly, the nanoemulsions were filtered through an 8 µm cellulose nitrate filter (Sartorius, Göttingen, Germany) to remove potential impurities [41]. For the preparation of CUR/QU-loaded NE (CQ NEdif-), the 3 mL of acetone:ethanol (60:40, *v/v*) was replaced by the mixture of the solutions of CUR (10 mg/Ml–1.5 mL) and QU (10 mg/mL–1.5 mL) in acetone:ethanol (60:40, *v/v*) (Table 1).

### 2.4. HPH Method Combined with the Phase Inversion Temperature Technique

The NEs prepared by HPH method were formed via high-energy emulsification followed by HPH of the mixture of water phase and oil phase [42]. In brief, to prepare the aqueous phase, the surfactant PEG 660-stearate was dissolved in ultrapure water (1.5% *w/v*). The oil phase containing castor oil, Lipoid^®^ purified fish oil (DHA/EPA), and egg lecithin (Lipoid E80^®^) was maintained for 30 min at 68 °C under magnetic stirring at 1500 rpm. Then the aqueous phase (60 mL), heated to 80 °C under magnetic stirring at 1500 rpm for 2 min, was added to the oil phase. After adding the aqueous phase to the oil phase, the dispersion was homogenized for 2 min using a mechanic high-performance dispersing device (Ultraturrax TP 18/10–10N, IKA-Werke GmbH, Staufen, Germany) at 14,500 rpm for 2 min to form the pre-emulsion. Finally, the pre-emulsion was passed through a high-pressure homogenizer (PandaPLUS 2000 Laboratory Homogenizer, GEA Niro Soavi, Parma, Italy) for 13 cycles of 20 s each at 1000 bar, totaling 4 min and 20 s to obtain the blank formulation (NE-). For the preparation of the formulation containing CUR and QU, the two natural compounds were added as powders to the organic phase and maintained under heating (68 °C) and stirring (1500 rpm) for 30 min (Table 1). This original formulation (CQ NE-) was modified to also obtain cationic NEs (CQ NE+). CQ NE+ were prepared by adding cetalkonium chloride (0.0175%, *w/v*) to the aqueous phase and heated until 68 °C with the surfactant, due to its hydrophilic character. To prepare the NEs containing the gelling agent, Gelzan™ CM (0.5%, *w/v*) was added to the formulation after the preparation process and kept under magnetic stirring at 700 rpm at 68 °C for 5 min.

### 2.5. Physico-Chemical and Morphological Characterization of NEs

#### 2.5.1. Stability Studies of NEs and Free CUR and QU

The stability of the proposed formulations was evaluated for visual appearance and content of CUR and QU over a period of one month at three different temperature conditions (4, 22, and 40 °C). In addition, there was performed a stability evaluation of free CUR and free QU in phosphate-buffered saline pH 7.4:PEG 400 (90:10) during 240 min at 37 °C.

#### 2.5.2. Size and Zeta Potential Measurements

The particle size and zeta potential of the NEs were determined by dynamic light scattering and laser doppler anemometry, respectively, using a Zetasizer Nano Series (Malvern Pananalytical, Malvern, UK) as reported in detail in previous studies [42].

#### 2.5.3. Morphological Evaluation of NEs

The morphology of NEs was observed using a transmission electron microscope (TEM) (JEOL 1400, Indianapolis, IN, USA). A drop of the NEs was diluted suitably (1:1000 with Milli-Q^®^ water) and deposited on a copper grid coated with carbon (200 mesh, Koch instrumentos científicos, SP, Brazil), followed by addition of 20 µL of uranyl acetate 2% *w/v* solution. Images were captured using the TEM operated at 80 kV and 30,000× magnification, for the formulation CQ NE- prepared by HPH, and 200,000× for the formulation CQ NEdif- prepared by HSD method combined with the phase inversion temperature technique.

#### 2.5.4. Determination of CUR and QU Concentration in the NEs by HPLC

The CUR and QU content was analyzed using a HPLC through an analytical method previously developed and validated [42]. 

#### 2.5.5. Determination of the Recovery and Encapsulation Efficiency

The CUR and QU recovery was calculated as being the percentage of the total drug concentration found in the suspensions in relation to the initially added amount. The encapsulation efficiency (%) was assessed as the difference between the total concentration of CUR/QU of the nanocarrier and the ultrafiltrate concentration. The filtrate was obtained by an ultrafiltration/centrifugation method of an aliquot (500 µL) of the NEs using an Ultrafree-MC^®^ (10,000 Da MWCO, Millipore, Bedford, MA, USA) in a centrifuge Sigma 3K30 (30 min at 10,000× *g*, San Luis, MO, EUA). All samples were analyzed in triplicate using a HPLC through an analytical method previously developed and validated [42].

#### 2.5.6. Evaluation of the Formulation Viscosity and Gelation of the Formulation in the Presence of Simulated Nasal Fluid in Vitro

In a beaker glass, 2 mL of the NEs was homogenized using a glass stirring rod for 1 min in simulated nasal fluid (SNF), aqueous solution pH 6.4 containing NaCl 8.766 mg/mL, KCl 2.98 mg/mL, NaH_2_PO_4_ 0.8998 mg/mL, Na_2_HPO_4_ 0.4258 mg/mL, and CaCl_2_·2H_2_O 0.5549 mg/mL) [43] in the volume proportion 1:2 (SNF:NE) in order to mimic the exposure of the formulation to the SNF in vivo, and the gelation of the formulations was observed. The viscosity of the formulations after exposure to SNF was then evaluated using a Rheostress Rheometer (HAAKE RS 150, Karlsruhe, Germany) with temperature controller (HAAKE DC 50, Karlsruhe, Germany) by varying the strain rate between 0.1 and 60 s^−1^, for 300 s at 37 °C, using a plate-cone-type sensor with a slope of 2° (C60) and a gap of 0.104 mm.

#### 2.5.7. Determination of Mucoadhesive Potential in Vitro

Mucoadhesion characteristics of the developed formulations, after and before the exposition to SNF, were evaluated by using an adaptation of a previously reported method [44], employing a texture analyzer equipped with a 5 kg load cell capacity (TA.XT plus^®^, Stable Micro Systems Ltd., Godalming, UK). Freshly porcine nasal mucosa was carefully removed from nasal turbinates by an incision along nasal septum from pig’s head, obtained from a local slaughterhouse, as described by Hägerström & Edsman [45]. SNF was prepared with mucin 8% (*w/v*). The nasal mucosa was maintained at 37 °C during the mucoadhesive measurement. The formulations (200 µL) were kept in the lower platform of the instrument and the nasal mucosa model in the upper movable probe (mucoadhesion ring with 10 mm diameter). A contact time of 60 s with the application of 9.8 mN of load force and then, in order to measure the adhesion forces developed, a traction speed of 0.5 mm/s were employed. The maximal force (mN) required for the detachment of samples from nasal mucosa was used to compare the formulations’ mucoadhesive properties.

#### 2.5.8. In Vitro Release Studies

For the drug release experiments, 1 mL of the NEs was placed into a dialysis bag (MWCO 10,000 Da). The dialysis bag was placed into a beaker containing 250 mL of PEG 400:distilled water (20:80, *v/v*; pH 4.0). The experiments were carried out in sink conditions. The solubilities of the CUR and QU in the dissolution medium are 0.099 mg/mL and 0.028 mg/mL, respectively. The release medium was maintained at 37 °C under magnetic stirring at 70 rpm. Samples of the medium were taken after 0.5, 1, 2, 4, 6, 8, 24, 48, and 72 h. The release medium was immediately replaced with the same amount of pre-warmed fresh medium. The samples were analyzed by HPLC under the conditions previously described. All experiments were carried out in triplicates. The samples were protected from light throughout the experimental procedure.

The cumulative amounts of CUR and QU released from the tested NE (in %) were plotted against time (h). For kinetics evaluation of the release profiles, the data were fitting to the model dependent of zero-order, first-order, and Higuchi, as follows:

(1) $$Q=Q_{0}+\mathrm{Kt}\;\;\;\;\;\;\;\;\;\;\;\;\;\;\;\;\;\;\;\;\;\;\;\;\;\;\;\;\;\;\;\;\;\;\;\;\;\;\;\;\left(\mathrm{Zero}\;\mathrm{order}\;\mathrm{model}\right)$$

(2) $$\mathrm{lnQ}={\;\mathrm{lnQ}}_{0}-\mathrm{Kt}\;\;\;\;\;\;\;\;\;\;\;\;\;\;\;\;\;\;\;\;\;\;\;\;\;\;\;\;\;\;\;\;\;\left(\mathrm{First}\;\mathrm{order}\;\mathrm{model}\right)$$

(3) $$Q\;=\frac{K1}{2t}\;\;\;\;\;\;\;\;\;\;\;\;\;\;\;\;\;\;\;\;\;\;\;\;\;\;\;\;\;\;\;\;\;\;\;\;\;\;\;\left(\mathrm{Higuchi}\;\mathrm{model}\right)$$

where Q is the amount of drug released in time t, Q_0_ is the initial concentration of the drug, and K is the model release constant.

#### 2.5.9. Ex Vivo Permeation and Retention Studies 

The permeation tests were performed using vertical Franz-type diffusion cells (2.268 cm^2^ surface area) equipped with freshly excised porcine nasal mucosa. The porcine nasal mucosa was obtained from a slaughterhouse authorized by the Ministry of Agriculture (Frigorífico Bonsul, Pelotas, RS, Brazil). Before the experiment, the porcine nasal mucosa was maintained for 30 min in SNF pH 6.4 [43].

The experiments were performed with the different developed nanocarriers. For the receptor solution, a SNF:PEG 400 (70:30, *v/v*; pH 6.4) mixture was used in order to maintain *sink* conditions at 37 ± 1 °C in a thermostatic bath with continuous magnetic stirring at 650 rpm for 12 h. Samples were withdrawn from the receptor compartment after 0.5, 2, 4, 8, 10, and 12 h of assay and immediately quantified by HPLC.

After 12 h, the porcine nasal mucosa was removed from the Franz apparatus, gently dried with a cloth, and washed with methanol to remove the excess formulation. To evaluate the retention of CUR and QU in the tissue, the exposed region of the porcine nasal mucosa was minced with a scalpel and placed in a volumetric flask with methanol, sonicated in an ultrasonic for 30 min, and stirred overnight. It was then filtered through a 0.45 µm membrane (Millipore Corporation, Billerica, MA, USA) and immediately quantified using HPLC.

#### 2.5.10. Worm Maintenance, Treatment, and Survival Test 

*C. elegans* Bristol N2 (wild type) was provided by the Caenorhabditis Genetic Center (CGC, University of Minnesota, Minneapolis, MN, USA). The strain N2 was grown at 20 °C on nematode growth media (NGM) plates (1.7% agar, 2.5 mg/mL peptone, 25 mM NaCl, 50 mM KH_2_PO_4_ pH 6.0, 5 μg/mL cholesterol, 1 mM CaCl_2_, 1 mM MgSO_4_) with fresh *Escherichia coli* OP50 as food source [46]. For each experiment, synchronized populations were obtained by disruption of gravid adults. Worms were grown to the L4 larval stage on NGM/OP50-seeded plates.

The young adult stage of *C. elegans* wild type was exposed to different concentrations of CUR and QU developed nanocarriers (CQ NEdif-, CQ NE+, and CQ NE-). In the survival test, treatment was performed with about 100 worms per group in M9 buffer (3 mg/mL of KH_2_PO_4_, 6 mg/mL of Na_2_HPO_4_, 5 mg/mL of NaCl, and 1 mL of 1 M MgSO_4_ in H_2_O). After 2 h, the worms were washed 3 times and transferred to NGM plates seeded with *Escherichia coli* OP50. After 24 h at 20 °C, survival evaluations were performed. As controls, the corresponding blank NE formulations and M9 buffer were used. 

The survival assay was performed following the protocol previously described, with some modifications [46]. About 100 nematodes per group were evaluated for viability under a Nikon E200 microscope (Tokyo, Japan). Animals that reacted to a mechanical stimulus were classified as alive and non-responding animals were classified as dead. Results were expressed as percentage of survivors. Analyses were performed in three independent trials. 

## 3. Results

### 3.1. Size and Polydispersity Index (PDI)

The formulations containing CUR and QU prepared by HSD (CQ NEdif-) showed a size of approximately 23 nm with a PDI of 0.300 and zeta potential of about −15 mV. In the case of the formulations obtained by HPH, the CQ NE- had a size of approximately 119 nm, PDI of 0.202, and zeta potential about −22 mV. After the addition of the gelling agent, CQ NEgel formulation presented a size of approximately 244 nm, PDI of 0.240, and zeta potential about −29 mV. Finally, for the cationic NEs (CQ NE+) the size was approximately 113 nm, PDI of 0.21, and zeta potential about +7 mV. Along with the measurements of the CQ formulations, the results for control NEs (NEdif-, NE-, and NE+), prepared without the active compounds, are reported in Table 2.

### 3.2. Stability Evaluation of NEs and Free CUR and QU

The proposed formulations showed good stability and no evident phase separation during the time of the test. In addition, the amount of CUR and QU in the formulations was monitored over a period of 30 days at three different temperature conditions (4, 22, 40 °C) (Table 3). The stability of free CUR and free QU was showed in Table 4.

CQ NEdif-, CQ NE-, and CQ NE+ at temperatures of 4 °C, 22 °C, and 40 °C, for 1 month, showed that the emulsions remained physically stable (without phase separation). The analysis of CUR and QU content inside the nanocarriers showed that the CQ NEdif- formulation was able to guarantee the integrity of CUR and QU at the three temperatures tested. However, in the CQ NE- formulation, CUR showed degradation on day 30 at temperatures of 22 and 40 °C, while QU only showed degradation at 40 °C but already from day 7. The CQ NE+ formulation was able to maintain the CUR integrity up to 30 days at 4 °C. However, at 22 °C and 40 °C CUR showed a significant decrease in concentration on day 30. The QU present in the positive formulation showed degradation at 40 °C already from day 7 of the experiment. However, the formulation was able to maintain the concentration of this compound until the end of the experiment at 4 and 22 °C. The free CUR and free QU evaluated at 37 °C showed a high degradation. Around 55% of CUR was degraded after 240 min, while the QU demonstrated a higher degradation, around 90% in the first 60 min (Table 4).

The stability evaluation of the formulations CQ NEdif-, CQ NE-, and CQ NE+ at temperatures of 4 °C, 22 °C, and 40 °C, for 1 month, showed that the NEs remained physically stable (without phase separation). The analysis of QU contents inside the nanocarriers showed stability for all the formulations at 4 °C and 22 °C during the period of the experiment. The analysis of CUR contents in the nanocarriers showed stability for all the formulations at 4 °C, showing some degradation after day 15 at 22 °C in the CQ NE-. At 40 °C, the concentration of CUR and QU was reduced over time for the CQ NE- and CQ NE+. The free CUR and free QU evaluated at 37 °C showed a high and rapid degradation. Around 55% of CUR was degraded after 240 min, while QU demonstrated an even higher degradation of about 90% in the first 60 min.

### 3.3. Determination of Drug Content, Recovery and Entrapment Efficiency

The validated HPLC method used [42] allowed us to determine the drug content, recovery, and entrapment efficiency of the developed NEs (Table 5). The formulations containing CUR and QU demonstrated that it was possible to encapsulate, on average, ~0.66 mg/mL of CUR and ~0.69 mg/mL of QU in the negative NEs prepared by HSD, ~0.72 mg/mL of CUR and ~0.61 mg/mL of QU in the negative NEs prepared by HPH, ~0.72 mg/mL of CUR and ~0.61 mg/mL of QU in the negative gel NEs prepared by HPH, and ~0.71 mg/mL of CUR and ~0.62 mg/mL of QU in the positive NEs prepared by HPH.

### 3.4. Transmission Electron Microscopy (TEM)

**Figure 1:**
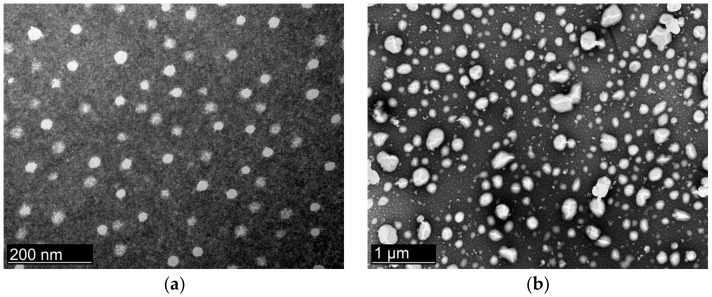
Transmission electron micrographs of the NEs: (**a**) CQ NEdif-, (**b**) CQ NE-. The images were obtained 10 days after the preparation of the formulations.

The TEM micrographs done by negative stain illustrated the size and morphology of the NEs produced with both techniques (Figure 1). The nanocarriers showed to be spherical in shape, with the dimension and polydispersity index in good agreement with the results of the analyses carried out by DLS. The particle size of CQ NEdif- and CQ NE- appears reliable with the results obtained by DLS.

### 3.5. Evaluation of Viscosity and Mucoadhesive Potential In Vitro

**Figure 2:**
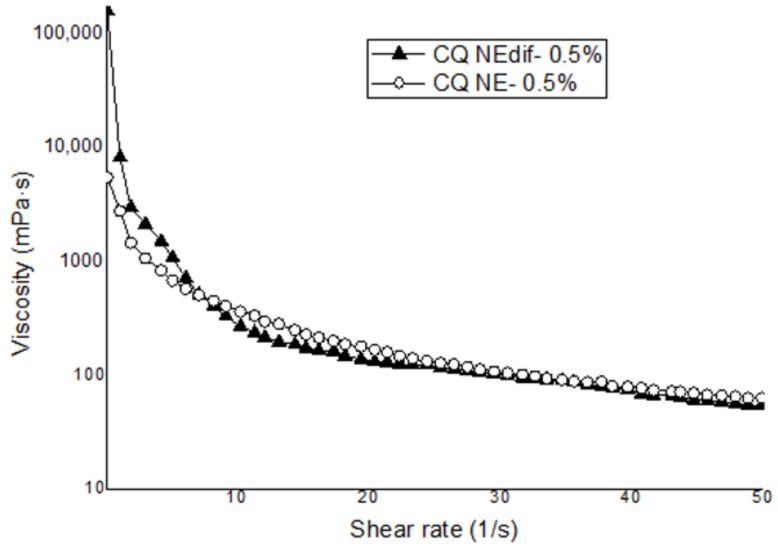
Evolution of the apparent viscosity versus shear rates for the NEs prepared with 0.5% of gelling agent. CQ NEdif- and CQ NE- exposed to the SNF.

A previous evaluation of different concentrations of gelling agent at the concentrations of 0.1, 0.3, and 0.5% (data not shown) was performed, and we opted to use the concentration of 0.5% of the gelling agent in this work due to the better stability of this formulation. The mucoadhesion results from the formulations prepared by HSD and HPH containing 0.5% of gelling agent are shown in Table 6. The formulations containing CUR and QU prepared by the two proposed techniques with 0.5% of gelling agent showed non-significantly different mucoadhesive strengths. Evaluation of the apparent viscosity versus shear rates demonstrated that formulations prepared by both method, HSD and HPH, when the same amount of gelling agent was added, showed similar non-Newtonian behaviors, with shear thinning characteristics (Figure 2).

### 3.6. In Vitro Release Studies

**Figure 3:**
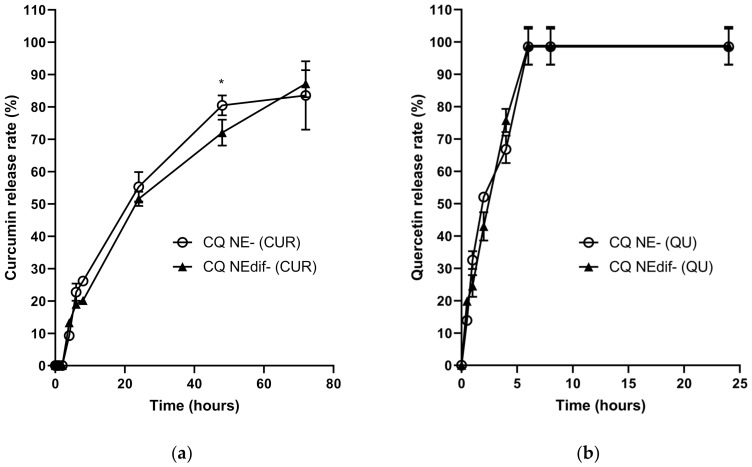
Cumulative percentage of CUR (**a**) and QU (**b**) released from the CQ NE- and CQ NEdif-. * *p* = 0.005 comparing CQ NE- (CUR), and CQNEdif- (CUR).

Figure 3 shows the in vitro release profiles of CUR and QU from the different nanocarriers (CQ NEdif-, CQ NE-) in PEG 400:distilled water (20:80, *v/v*; pH 4.0) at 37 °C. The release data comparison showed a similar behavior between CQ NEdif- and CQ NE-, since only in 48 h of CUR release profile from CQ NE- was there observed a statistical difference. However, it can be observed that CUR release occurred in a sustained manner in comparison to the QU release, considering that CUR was released continuously for up to 72 h without the existence of a plateau. With data fitting in mathematical models, it was shown that CUR and CQ were released from CQ NE- and CQ NEdif- following a first-order kinetics, with correlation coefficient very close to 1.0 (Table 7). The first-order kinetics indicates that the drug release rate is directly proportional to the remaining drug in the carrier; that is, the higher the concentration of drug within the carrier, the higher is the release rate.

### 3.7. Permeation and Retention of CUR and QU through Porcine Nasal Mucosa

**Figure 4:**
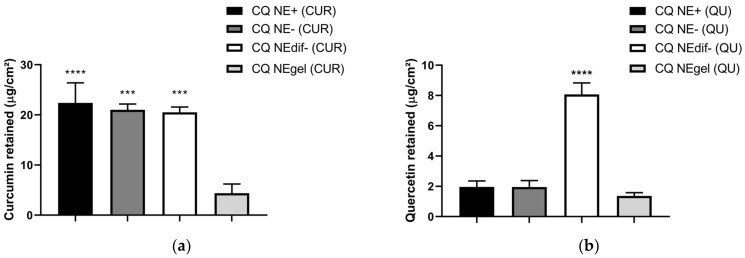
(**a**) Mass of CUR retained in porcine nasal mucosa after 12 h of permeation test in Franz-type diffusion cell. The CUR was extracted with methanol overnight. **** *p* < 0.001 and *** *p* = 0.001 compared to CQ NEgel (CUR). (**b**) Mass of QU retained in porcine nasal mucosa after 12 h of permeation test in Franz-type diffusion cell. The QU was extracted with methanol overnight. **** *p* < 0.001 compared to CQ NE+, CQ NE-, and CQ NEgel.

The results of CUR retained in porcine nasal mucosa normalized by surface area (µg/cm^2^) are shown in Figure 4a. It can be seen that there was a similar retention between the formulations, CQ NE- (21.01 µg/cm^2^) and CQ NEdif- (20.50 µg/cm^2^), after 12 h. The results of QU retained per area (µg/cm^2^) in porcine nasal mucosa are shown in Figure 4b. It can be seen that there was better retention from the formulation CQ NEdif- (8.07 µg/cm^2^) when compared to the CQ NE- (1.95 µg/cm^2^) formulation after 12 h. 

**Figure 5:**
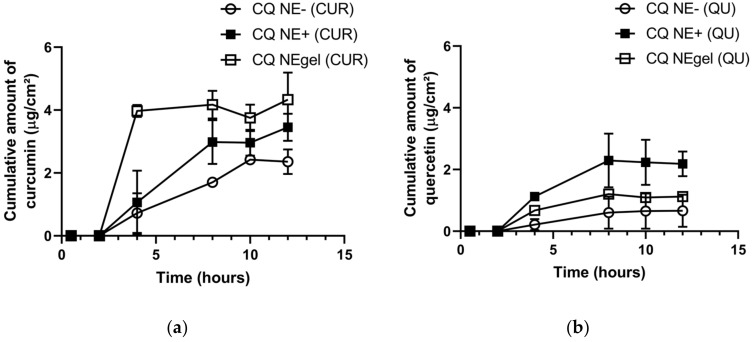
Cumulative amount (µg/cm^2^) of CUR (**a**) and QU (**b**) permeated from CQ NE-, CQ NE+, and CQ NEgel, through porcine nasal mucosa, in polyethylene glycol 400:SNF (20:80, *v/v*; pH 6.4) at 37 °C.

The ex vivo permeation of CUR and QU across porcine nasal mucosa using PEG 400:SNF (70:30, *v/v*; pH 6.4) as receiving medium at 37 °C showed different behaviors for CUR/QU permeations from the developed nanocarriers. The nanocarrier prepared by the HSD made possible the detection of higher amounts of CUR and QU in the receptor chamber since the first 2 h and 4 h of the experiment, respectively. However, a concentration plateau for both compounds was observed in the subsequent times (Supplementary Materials Table S1). On the other hand, the negative formulation produced by HPH showed a potential for sustained permeation until the tenth hour (CUR), showing an increase of 1.5× from the eighth to the tenth hour. The behavior of the formulation obtained by HPH led to the selection of this formulation for the subsequent studies and optimization, such as the development of a cationic formulation and an in situ gelling formulation. Figure 5 compares the permeation behavior of the formulations prepared by HPH. The cationic (CQ NE+) and the in situ gelling formulation (CQ NEgel) produced by HPH showed a potential for sustained permeation until the eighth hour for both CUR and QU. The permeation of CUR was more important with the gel formulation (CQ NEgel), probably because of the effect related to the viscosity of the formulation, while, interestingly, for QU it was the cationic formulation (CQ NE+) that worked better. These data indicate that the mechanism of transport is not the same and that the characteristics of the two natural substances determine a different behavior with the formulations proposed.

### 3.8. Caenorhabditi Elegans Lifespan Assay

**Figure 6:**
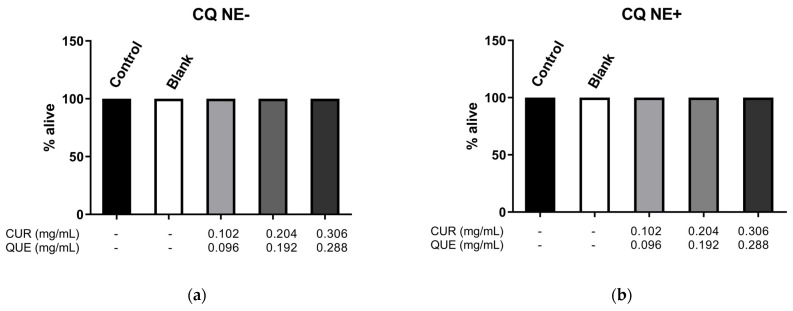

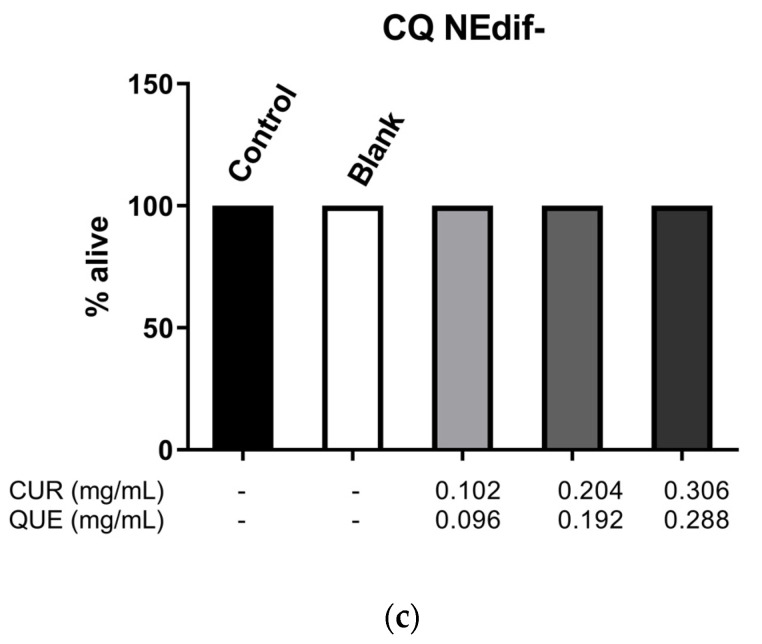
Survival rate of N2 *C. elegans* strain after 2 h exposition to different concentrations of CQ NE- (**a**), CQ NE+ (**b**), and CQ NEdif- (**c**). Data from three independent experiments.

The *C. elegans* lifespan assay was performed to evaluate the toxicity of the developed nanocarriers. The worms were treated with sequential dilutions of the NEs to obtain various concentrations of CUR and QU. The treatments were performed with all types of nanocarriers developed in this work (Figure 6), CQ NE+ (Figure 6a), CQ NE- (Figure 6b), and CQ NEdif- (Figure 6c). Treatments showed no significant differences compared to the controls (blank formulations and M9 buffer). The nanocarriers loaded with CUR and QU developed in this work did not cause any toxicity in *C. elegans*.

## 4. Discussion

The main results of this study are the evidence that it is possible to encapsulate a large quantity of CUR and QU in ω-3 fatty acids containing NEs using a small amount of surfactant and different production techniques. The developed NEs showed different behaviors in the permeation/retention experiments, which could indicate a possible increase in the permeation of CUR and QU through the nasal mucosa, with important characteristics that may be useful for the treatment of brain diseases. The developed NEs in this work are innovative because of the association of the natural compounds CUR, QU, and PUFAs in NEs to the brain delivery by the IN route using a reduced amount of surfactant.

Administration of CUR and QU has been proposed for prevention and treatment of the oxidative stress that is directly linked to ND [11,47]. Abdel-Diam and collaborators (2019) studied the effect of CUR and QU oral administration, alone or in association, to albino rats exposed to diazinon, an organophosphorus insecticide that induces neurotoxicity. They concluded that the treatment with CUR and QU individually or in combination reduced the inflammation and improved liver and brain antioxidant status, reducing oxidative stress levels induced by diazinon exposure. Noteworthily, the amelioration of oxidative stress and improvement of antioxidant capacity were greater when these compounds were administered in combination compared to the single compound administrations [48].

Studies have been developing nanocarriers to the central nervous system through the IN route in order to take better advantage of the properties of these compounds in the therapeutics. Madane and Mahajan developed nanostructured lipid carriers containing CUR, produced by HPH, for treatment of brain cancer through the IN route. They demonstrated, through biodistribution studies using male Wistar rats, a greater drug absorption in brain after IN administration of the studied compound, proving that it is possible to direct the CUR to the brain after IN administration to Wistar rats [49]. It is important to highlight that the formulations developed in our work were enhanced with the cetalkonium chloride and with the gelling agent, making possible the production of two different formulations that can improve the permeation and consequently the concentration of CUR and QU in the SNC. Given that CUR and QU were capable of ameliorating the oxidative stress and improving the antioxidant capacity when administered together and enhanced the permeation using nanotechnology and administration by IN route, we believe that the association of these compounds with ω-3 PUFAs, such as DHA, and the administration by the IN route of a nanocarrier composed by these compounds can be an interesting alternative for brain disease treatment.

DHA is one of the most abundant ω-3 PUFAs in the brain, being concentrated in the phospholipid membrane, particularly at the synapses [34,50,51]. PUFAs contribute to neuroplasticity [52], prevent oxidative stress [53], and are pivotal for the generation of anti-inflammatory factors in the CNS [54,55]. For these reasons, PUFAs were selected to be added to the oil phase of the formulations instead of other possible oil components. 

Karthik and Anandharamakrishnan developed a NE containing DHA, and the formulation was able to protect this ω-3 fatty acid against the oxidation responsible for chemical instability of this compound. The functional groups of DHA remained intact and did not undergo any modification of functional activity during the nanoemulsification process [56]. During the process, we heated the oil and the aqueous phases to minimum temperatures needed to dissolve and solubilize the components of the oil phase, aiming to preserve the PUFAs against thermal degradation, but enough to promote the change in the Solutol HS15^®^ conformation at the aqueous phase. 

In the present work, we chose to realize the NEs using two methods: HSD associated with phase inversion temperature method and HPH. Stability tests performed with the proposed formulations demonstrated that the lipid nanocarriers were able to enhance the chemical stability of CUR and QU. The formulation CQ NEdif- was able to protect these compounds against degradation during the 30 days of the study at all the temperatures tested (4 °C, 22 °C, and 40 °C). However, the formulations prepared by the HPH method demonstrated a slightly reduced ability to maintain the stability of nanoencapsulated compounds. We believe that the reduced capacity occurs due to the higher amount of oil in these formulations. The oil oxidation can be responsible for the higher degradation of the nanoencapsulated compounds. Nevertheless, in comparison to the free CUR and free QU, the developed nanocarriers were efficient in preserving these compounds from degradation. In the free form, QU was 90% degraded in 60 min, while CUR showed a lower degradation; around 55% was degraded after 240 min.

The lipid nanocarriers permitted the incorporation of similar quantities of CUR (~0.632 mg/mL) and QU (~0.702 mg/mL) for both techniques with a high entrapment efficiency (>99%). These results, along with the size of approximately 20 nm by the HSD, are in accordance with the results obtained from the NE prepared by HSD only with CUR or QU, which was able to incorporate approximately 1.5 mg/mL of each compound, with sizes around 22 nm [18,57]. Regarding the HPH method, Busmann and collaborators developed a NE using 4% of Solutol HS15^®^ as a surfactant to obtain formulations of approximately 150 nm [58]. Herein, we obtained formulations of ~115 nm using only 1.6% of surfactants. The morphological analysis of NEs indicates that the particles show some faceted part, and the contrast of some particles, observed at a higher magnification, is reliable as nanocapsule morphology. This is in line with a previous study of our group that showed small and wide angle X-ray scattering (SAXS) results indicating an internal structure of a nanosized emulsion loading QU with a core-shell spherical structure for the CQ NEdif- [59].

To better understand the behavior and the drug release of the nanoformulations containing CUR and QU developed in this work, a comparison between them using in vitro dialysis release was accomplished. The diffusion test demonstrated for both formulations a controlled release of CUR over 72 h and a fast release in the first 6 h for QU. The CUR release results are in accordance with the those obtained by Muntimadugu and collaborators in a study where nanocarriers, with less than 200 nm encapsulating a lipidic compound, demonstrated an in vitro sustained release indicating a prolonged residence time of the drug at the targeting site [60]. The fast release of QU followed by a sustained release of CUR is an interesting result, because with this behavior the active compounds can act in a complementary way, when in joint administration. Firstly, QU is released, followed by CUR, thus covering a longer treatment time. This fact may occur because QU (LogP 1.82) is less lipophilic than CUR (LogP 1.94), being released faster from the oil phase. Higher values of LogP indicate lipophilicity and higher affinity for the lipid phase [61]. For release kinetics evaluation, the in vitro release data were fitted to three different models, zero-order, first-order, and Higuchi. The results indicated that the first-order kinetics was the best fitting for all the tested formulations (Table 7), demonstrating that the release was controlled by the amount of drug remaining in the system and not by the structure of the carrier. So, the release of the active compounds from CQ NEdif- and CQ NE- occurred in proportion to the amount of CUR and QU remaining inside the nanocarrier.

Another assay performed to understand the behavior of CUR and QU from the developed nanocarriers was the permeation/retention test in porcine nasal mucosal tissue, using Franz-type diffusion cell. In the permeation evaluation, it was possible to detect CUR and QU in the acceptor chamber and the mucosa. The porcine nasal mucosa was used to test the passage of drugs through the nasal mucosa, aiming to target the compounds to the central nervous system [62], permitting an initial evaluation of formulations, and consequently decreasing the use of experimental animals [63,64]. The SNF:PEG 400 (70:30, *v/v*; pH 6.4) was selected as a fluid of the acceptor chamber, since it has the capability to solubilize both CUR and QU. Between the formulations developed in this work (CQ NE-, and CQ NEdif-), the results showed differences in the behavior of the permeation and retention. The results obtained by the CQ NE-, produced by HPH method, showed a controlled release until the 12th hour (CUR), which is interesting for the possible treatment of ND. However, for the CQ NEdif-, this controlled permeation potential was not observed, even if a more efficient permeation, due to the smaller size of this formulation, can be achieved. For the intended type of treatment, a better permeation of these natural compounds is the main goal, and the sustained permeation is more advantageous than a faster permeation.

The difference in the retention of CUR and QU probably occurred due to the difference in lipophilicity of these compounds. CUR showed a water solubility of 0.00575 mg/mL, while QU showed greater hydrophilicity, 0.261 mg/mL, thus approximately 45 times more soluble in water. Compared to the QU, the CUR showed, from all tested formulations, that a higher concentration in the porcine nasal mucosa was possible as a consequence of its lipophilicity. Between the negative developed formulations, a difference was observed only for the retention of QU, being higher for the CQ NEdif-. Nevertheless, the permeation achievements are more important than the retention of the compounds in the mucosa for the desired treatment. Therefore, the behavior of controlled permeation is preferable compared to rapid permeation, even if the last occurs in greater concentration and despite the retention values.

These observations, added to the possibility of scaling the production method to industry and of avoiding organic solvent use in the preparation process, led to the selection of CQ NE- for the subsequent studies and improvements, such as the development of a cationic formulation and an in situ gelling formulation. Furthermore, no significant difference was detected between the nanocarriers prepared by HSD and HPH in terms of viscosity after the addition of the gelling agent, which supports the choice made based on the other criteria.

The development of the cationic formulation and the in situ gelling formulation were considered to enhance the penetration through the nasal mucosa. To produce a cationic formulation, cetalkonium chloride was added to the formulation, and the value obtained was about +7 mV, in accordance with the strategy used by Daull et al. [65], which obtained a charge of +7.9 mV due to the addition of this cationic agent. With the conversion of the formulation charge from negative to positive, we were able to enhance the permeation of CUR and QU. This is in agreement with the results found by Chen et al. that evaluated positively charged, negatively charged, and untreated hydroxyapatite nanoparticles over the ability to penetrate osteoblasts. They concluded that the nanoparticles with positive charge had higher uptake into cells compared to nanoparticles with negative charge, which may be certified to the attractive or repulsive interaction between the negatively charged cell membrane and positively/negatively charged nanoparticles [66]. 

Another technique to enhance the bioavailability of the compounds by IN administration is the use of in situ gelling agents, aiming to overcome the presence of the nasal mucociliary clearance in IN administration. Galgatte and collaborators developed a mucoadhesive in situ gel to extend formulation residence time at the nasal site and facilitate the uptake of the drug to the brain. As a result, they concluded that the in situ gel prepared with deacetylated gellan gum enhanced nasal residence time and permitted an efficient nose-to-brain transport [67]. In another study, Salem and collaborators developed a nasal mucoadhesive nano-emulgel for the direct brain targeting of resveratrol. The developed formulations were administered via the IN route and compared to resveratrol suspension oral administration to Wistar rats. The authors concluded that cerebral bioavailability was increased by the IN nano-emulgel formulations [68]. Based on the abovementioned works, we decided to combine the deacetylated gellan gum and nanotechnology techniques to develop NEs with the addition of this gelling agent, aiming to enhance the residence time of the formulation in the nasal cavity and possibly the bioavailability of the compounds CUR and QU in the brain. The data obtained from the viscosity analyses demonstrated that the formulation tested showed non-Newtonian behavior, with a shear-thinning characteristic. The formulation with in situ gelling agent (CQ NEgel) was able to enhance the permeation of CUR when compared to the formulations prepared by the same method with negative (CQ NE-) and positive (CQ NE+) charges.

Lastly, we also tested the toxicity of the formulations in an animal model, *C. elegans*, which are eukaryotic nematode organisms like mammals and present about 60–80% of genes homologous with humans [69]. The invertebrate organisms model is a possible bridge between cell cultures and mammals [70]. This model is an alternative and complementary system for deciphering ND etiologies and to investigate possible new drugs. *C. elegans* transgenic strains that express Ab allow studies of Alzheimer’s Disease, and transgenic strains of Parkinson’s Disease have been successfully used to study Parkinson’s Disease-like pathologies and behaviors [71,72]. This small free-living soil nematode is an important experimental model in research areas such as molecular biology, toxicology, and pharmacology and has been successfully used for decades [73,74]. It has been used for drug screenings due to its relatively short lifespan (about 20 days), small size, and rapid life cycle [75]. The animal presents easy genetic manipulation and fully identified genome [76]. We performed a survival test using *C. elegans*, and the obtained results demonstrated no toxicity for all tested formulations (CQ NEdif-, CQ NE+, and CQ NE-) in different concentrations, concluding that the nanocarriers developed in this work are safe for further experiments.

Nanocarriers with low amounts of surfactant are much better tolerated. The two techniques proposed in this work allowed the production of NEs with suitable sizes using just a low amount of surfactant and co-surfactant. We used only 1.6% of surfactant in the formulation. This fact makes the nanocarrier safer and lower in toxicity, as confirmed in this work with the absence of worm (*C. elegans*) deaths in the concentrations of NEs to which these animals were exposed.

## 5. Conclusions

The results demonstrated that it was feasible to develop different types of NEs with ω-3 fatty acids containing significant amounts of CUR and QU using a small amount of surfactants and showing proper physicochemical properties. The formulations showed great stability, especially at 4 °C, and no toxicity. Considering the two preparation methods tested, HPH shows advantages, and the CQ NE- obtained demonstrated potential for sustained release, making this the chosen formulation for the further experiments and improvements, such as the development of a cationic formulation and an in situ gelling formulation. Regarding mucoadhesive potential, we observed similar strength results for formulations obtained by both methods, supporting that the choice for gelling agent addition could be made based on other criteria, such as the release profiles. Both modifications applied to the original HPH formulation were able to improve the permeation capacity for the two carried compounds. However, for retention tests, the improvement could not be seen, and there was even an inferior retained value for the gelling formulation. These retention results did not prevent the benefits for the main goal, which is permeation, to be achieved. In view of the fact that the proposed alterations promoted increases in permeation, they are relevant to be applied. However, to better understand the results, in vivo studies in rodents should be performed. 

## Figures and Tables

**Table 1:** Composition of the negative nanocarriers prepared through the HSD method (NEdif- and CQ NEdif-) and through HPH method (NE- and CQ NE-), both methods associated with the phase inversion temperature technique. Amount of CUR and QU in the formulation (±1.75 mg/dL in total).

Formulation	PEG 660 Stearate (% *w/v*)	Castor Oil (mg)	ω-3 Fatty Acids (mg)	Egg Lecithin (mg)	CUR (mg)	QU (mg)
CQ NEdif-	1.5	0.0750	0.0750	20	15	15
NEdif-	1.5	0.0750	0.0750	20	–	–
CQ NE-	1.5	2400	2400	1200	45	45
NE-	1.5	2400	2400	1200	–	–

**Table 2:** Size, PDI, and zeta potential of the NEs.

Preparation Method	Formulation	Size ± SD (nm)	Polydispersity Index ± SD (PDI)	Zeta Potential ± SD (mV)
Hot solvent diffusion	CQ NEdif-	23.03 ± 3.11	0.300 ± 0.10	−15.10 ± 2.35
	NEdif-	19.02 ± 1.30	0.260 ± 0.06	−25.30 ± 2.42
High-pressure homogenization	CQ NE-	119.43 ± 0.83	0.202 ± 0.02	−22.30 ± 0.15
	NE-	102.86 ± 1.80	0.183 ± 0.02	−25.70 ± 0.46
	CQ NEgel	244.80 ± 2.40	0.240 ± 0.03	−29.10 ± 0.40
	NEgel	134.87 ± 0.40	0.230 ± 0.02	−25.70 ± 0.20
	CQ NE+	113.00 ± 0.25	0.210 ± 0.01	+7.90 ± 0.24
	NE+	131.86 ± 0.8	0.246 ± 0.01	+6.40 ± 0.20

**Table 3:** Percentage (%) of the CUR and QU in the NEs (CQ NEdif-, CQ NE-, CQ NE+) during 30-day stability studies at 4, 22, and 40 °C.

Formulation		Temperature (°C)	Day 7	Day 15	Day 30
CQ NEdif-		4	97.81 ± 2.7	98.49 ± 1.3	99.59 ± 0.5
	CUR	22	96.99 ± 0.6	97.26 ± 4.0	98.49 ± 2.7
		40	98.77 ± 0.9	99.15 ± 1.0	99.10 ± 0.8
		4	96.21 ± 1.2	96.07 ± 2.7	99.72 ± 2.7
	QU	22	94.27 ± 1.3	93.45 ± 0.5	94.50 ± 1.3
		40	94.40 ± 0.6	96.04 ± 2.0	96.58 ± 1.7
CQ NE-		4	99.15 ± 1.3	99.66 ± 1.0	95.09 ± 3.3
	CUR	22	98.30 ± 6.7	97.29 ± 5.0	87.98 ± 3.3
		40	94.75 ± 3.3	95.09 ± 3.0	85.95 ± 1.6
		4	98.69 ± 0.4	99.10 ± 1.6	97.01 ± 3.3
	QU	22	94.37 ± 3.1	96.02 ± 4.9	96.35 ± 2.9
		40	85.09 ± 1.1	74.17 ± 1.6	64.73 ± 4.9
CQ NE+		4	99.53 ± 3.0	95.39 ± 3.3	96.47 ± 0.6
	CUR	22	94.63 ± 1.5	94.30 ± 0.3	91.94 ± 1.5
		40	92.94 ± 1.5	93.09 ± 0.6	87.88 ± 1.5
		4	96.02 ± 3.0	95.41 ± 0.6	95.60 ± 1.5
	QU	22	95.41 ± 3.2	95.10 ± 2.1	95.84 ± 1.3
		40	76.91 ± 3.0	57.33 ± 1.0	50.30 ± 3.0

**Table 4:** Percentage (%) of free CUR and free QU during 240 min in phosphate-buffered saline pH 7.4:PEG400 (90:10) stability study at 37 °C. Results are expressed as mean ± standard deviation.

Compound	0 (min)	60 (min)	120 (min)	180 (min)	240 (min)
Free CUR	100	91.8 ± 5.0	76.5 ± 9.0	59.6 ± 7.2	46.1 ± 9.4
Free QU	100	8.9 ± 1.5	7.9 ± 5.4	0.0 ± 0.0	0.0 ± 0.0

**Table 5:** Drug content, recovery, and entrapment efficiency of the nanoemulsions.

Formulation	Drug Loading QU (mg/mL)	Recovery (%)	Entrapment Efficiency (%)	Drug Loading CUR (mg/mL)	Recovery (%)	Entrapment Efficiency (%)
CQ NEdif-	0.66 ± 0.03	88.00 ± 4.0	>99	0.69 ± 0.02	92.00 ± 2.6	>99
CQ NE-	0.72 ± 0.01	96.00 ± 1.3	>99	0.61 ± 0.01	81.33 ± 1.3	>99
CQ NEgel	0.72 ± 0.01	96.00 ± 1.3	>99	0.61 ± 0.01	81.33 ± 1.3	>99
CQ NE+	0.71 ± 0.03	94.66 ± 4.0	>99	0.62 ± 0.02	82.66 ± 2.6	>99

**Table 6:** Mucoadhesive strength of the formulations developed by both techniques with CUR and QU (CQ) at the concentration 0.5% of the gelling agent.

	Force (mN)
CQ NEdif- 0.5%	7.59 ± 0.31
CQ NE- 0.5%	8.70 ± 1.10

**Table 7:** Kinetics parameters obtained from CUR and QU release profiles.

Formulation			Correlation Coefficient (r^2^)
CQ NE-	QU	First-order parameters	0.9648
	CUR	First-order parameters	0.9753
CQ NEdif-	QU	First-order parameters	0.9820
	CUR	First-order parameters	0.9957

## Data Availability

Data presented in this article is available on request from the corresponding author.

## Supplementary Materials

The following supporting information can be downloaded at: https://www.mdpi.com/article/10.3390/nano12071073/s1, Table S1. Ex vivo Permeation release of QU and CUR.
