# Supplementary material for: Curcumin and Quercetin-Loaded Lipid Nanocarriers: Development of Omega-3 Mucoadhesive Nanoemulsions for Intranasal Administration
Source: Nanomaterials (Basel). 2022 Mar 25;12(7):1073. doi: 10.3390/nano12071073 (PMC9000715; doi:10.3390/nano12071073)
Supplement: Supplementary file 1 [file nanomaterials-12-01073-s001.zip › nanomaterials-1625533-supplementary.pdf]

nanomaterials

Supplementary Materials

Curcumin and Quercetin-Loaded Lipid Nanocarriers: Development of Omega-3 Mucoadhesive Nanoemulsions for Intranasal Administration

Gustavo Richter Vaz [1], Mariana Corrêa Falkembach Carrasco [1], Matheus Monteiro Batista [1], Paula Alice Bezerra Barros [1], Meliza da Conceição Oliveira [1], Ana Luiza Muccillo-Baisch [1], Virginia Campello Yurgel [1], Francesca Buttini [2], Félix Alexandre Antunes Soares [3], Larissa Marafiga Cordeiro [3], Flavia Fachel [4], Helder Ferreira Teixeira [4], Juliana Bidone [5], Patrícia Diaz de Oliveira [6], Fabio Sonvico [2] and Cristiana Lima Dora [1,*]

Table S1. Ex vivo Permeation release of QU and CUR.

Formulation	Time (Hours)	Ex Vivo Permeation Release of QU (%)	Ex Vivo Permeation Release of CUR (%)
CQ NEdif-	0.5	0.00 ± 0.00	0.00 ± 0.00
	2.0	0.00 ± 0.00	8.28 ± 0.98
	4.0	1.12 ± 0.04	6.32 ± 1.11
	8.0	2.29 ± 0.87	7.91 ± 2.08
	10.0	2.23 ± 0.73	7.59 ± 1.45
	12.0	2.18 ± 0.40	6.91 ± 0.96
CQ NE-	0.5	0.00 ± 0.00	0.00 ± 0.00
	2.0	0.00 ± 0.00	0.00 ± 0.00
	4.0	0.21 ± 0.18	0.72 ± 0.63
	8.0	0.60 ± 0.52	1.70 ± 0.10
	10.0	0.65 ± 0.57	2.42 ± 0.02
	12.0	0.66 ± 0.52	2.36 ± 0.39
